# Dynamic Changes in the Renin-Angiotensin-Aldosterone System and the Beneficial Effects of Renin-Angiotensin-Aldosterone Inhibitors on Spatial Learning and Memory in a Rat Model of Chronic Cerebral Ischemia

**DOI:** 10.3389/fnins.2017.00359

**Published:** 2017-06-23

**Authors:** Xinwu Huang, Guozhou Lu, Guochun Li, Hua Li, Beihua Li, Jiazhen Yin, Shousong Cao

**Affiliations:** ^1^Department of Pharmacology, Southwest Medical UniversityLuzhou, China; ^2^Department of Pharmacy, Xichang People's HospitalXichang, China; ^3^The Affiliated Hospital of Traditional Chinese Medicine, Southwest Medical UniversityLuzhou, China

**Keywords:** cerebral ischemia, spatial learning and memory, apoptosis, renin angiotensin aldosterone system (RAAS), RAAS inhibitor, rat model of chronic cerebral ischemia

## Abstract

Renin-angiotensin-aldosterone system (RAAS) plays an important role in the regulation of blood pressure and brain function. Therefore, we studied the dynamic changes in the RAAS in the blood, cerebral cortex, and hippocampus and the effects of RAAS inhibitors on spatial learning and memory and hippocampal apoptosis in a rat model of chronic cerebral ischemia (CCI) established by bilateral ligation of the common carotid arteries of rats. The levels of renin, angiotensin II (Ang II), and aldosterone (ALD) in the plasma, and the homogenates of the left side of cerebral cortex and whole hippocampus of rats were detected on day 1, 3, 7, 14, 21, and 30 by radioimmunoassay. Spatial learning and memory and hippocampal apoptosis were evaluated on day 30 by Morris water maze test (navigation and space exploration tests) and terminal dexynucleotidyl transferase (TdT)-mediated dUTP nick end labeling (TUNEL) assay, respectively, after rats were orally administered with distilled water (DW), renin inhibitor aliskiren (30 mg/kg), Ang converting enzyme inhibitor enalapril (4 mg/kg), or Ang II receptor antagonist candesartan (2 mg/kg) daily for 30 days. The results showed that the levels of renin and Ang II were significantly higher but ALD fluctuated in the blood, cerebral cortex, and hippocampus in CCI rats compared to normal rats. However, aliskiren and enalapril could significantly decrease (*p* < 0.05) the levels of renin, Ang II and ALD in the blood, cerebral cortex, and hippocampus compared to DW treatment; while candesartan had similar effect on renin and ALD but no effect on Ang II in CCI rats. Furthermore, spatial learning and memory were significantly decreased but apoptosis in the hippocampus was obviously increased in CCI rats compared to normal rats (*p* < 0.05). However, aliskiren, enalapril, and candesartan were equally effective to improve spatial learning and memory and decrease apoptosis in the hippocampus. Therefore, RAAS plays an important role in the development of cerebral ischemia and RAAS inhibitors aliskiren, enalapril, and candesartan improve spatial learning and memory and protect brain injury by inhibiting hippocampal apoptosis in CCI rats.

## Introduction

The renin-angiotensin aldosterone system (RAAS) plays an important role in the regulation of plasma sodium concentration, extracellular volume, tissue perfusion, and blood pressure (Atlas, 2007). RSSA is activated when the blood volume and/or blood pressure are decreased (Beuschlein, 2013; Maron and Leopold, 2014). Renin is released by juxtaglomerular cells in the kidney into the circulation to cleave angiotensinogen which is synthesized and secreted by the liver to angiotensin I (Ang I) and further converted to angiotensin II (Ang II) by angiotensin-converting enzyme (ACE) located primarily in the pulmonary capillaries and renal endothelium (Paul et al., 2006; Yee et al., 2010; Friis et al., 2013). Ang II induces the release of aldosterone (ALD) from the zona glomerulosa in the adrenal cortex and is largely responsible for the long-term regulation of blood pressure (András and Hunyady, 2004; Hu et al., 2012; Bollag, 2014; Pacurari et al., 2014).

Studies have shown that the RAAS regulates the physiological and pathological activities in the brain (Hajjar et al., 2009). Numerous studies have revealed that Ang in the central RAAS is closely associated with learning and memory (Gard, 2002, 2004; Gard and Rusted, 2004; Ciobica et al., 2009). A study by Kumaran and colleagues suggested that Ang II played a key role in nerve cell death and memory loss during brain hypoperfusion (Kumaran et al., 2008). However, the dynamic change of RAAS in the brain ischemic injury has not been reported. Therefore, the alterations of RAAS induced by chronic cerebral ischemia (CCI) and the correlation between it and brain dysfunction need to be further studied.

Aliskiren (Tekturna) is a direct renin inhibitor for oral treatment of patients with essential hypertension (Anderson, 2007). Aliskiren binds with renin to block the conversion of angiotensinogen to Ang I (Politi et al., 2011). Enalapril (Vasotec) is a nonsulfhydryl ACE inhibitor for the treatment of patients with hypertension, symptomatic heart failure and chronic kidney failure (Ferguson et al., 1982; Vlasses et al., 1985; McMurray, 2010; He et al., 2013). Enalapril inhibits Ang I converted to Ang II to decrease the level of Ang II leading to less vasoconstriction and lower blood pressure in patients with hypertension (Sweet et al., 1983). Candesartan is a potent and selective Ang II receptor antagonist primarily used for the treatment of patients with hypertension. It binds tightly to and dissociates slowly from the Ang II type 1 (AT1) receptor to mediate the activities of Ang II and effectively reduce blood pressure in patients with hypertension (McClellan and Goa, 1998).

The cerebral cortex and hippocampus are very sensitive to ischemic injury. Hippocampus plays an important role in spatial memory (Shrager et al., 2007). We have found that it took about 30 days to affect spatial learning and memory and it would not recover in 2 months in CCI rats induced by bilateral ligation of the common carotid arteries (Huang et al., 2010). Thus, we used cerebral cortex and hippocampus and selected the 30 day time-course in the present studies. Apoptosis plays a key role in cerebral ischemia (Broughton et al., 2009). Study has shown that baicalin extracted from dry dicotyledonous skullcap root inhibited hippocampal apoptosis to improve spatial memory in the state of global cerebral ischemia in rats (Cheng et al., 2012). Our recent study also showed that saponins isolated and extracted from lychee seeds significantly improved spatial learning and memory and prevented neuronal injury by inhibiting apoptosis in a rat model of Alzheimer's disease (Wang et al., 2017). Therefore, we evaluated the inhibitory effect of RSSA inhibitors on apoptosis in the hippocampus in CCI rats.

In the present study, we established a rat model of CCI and evaluated the levels of renin, Ang II, and ALD in the blood, cerebral cortex and hippocampus of rats on day 1, 3, 7, 14, 21, and 30 after bilateral ligation of the common carotid arteries to study the dynamic changes. Furthermore, we also studied the effects of renin inhibitor aliskiren, long-acting ACEI enalapril, and Ang II AT1 receptor blocker candesartan on improvement of spatial learning and memory and protection of brain damage via inhibiting hippocampal apoptosis in CCI rats.

## Materials and methods

### Drugs and reagents

Aliskiren (lot number: SH140319) was purchased from Wuhan Sanhuan Pharmaceutical and Chemical Co., Ltd. (Wuhan, Hubei, China). Enalapril (lot number: 13110702) was purchased from Yangtze River Pharmaceutical Group, Jiangsu Pharmaceutical Co., Ltd. (Taizhou, Jiangsu, China). Candesartan (lot number: 130601) was purchased from Zhejiang Yongning Pharmaceutical Co., Ltd. (Taizhou, Zhejiang, China). Radioimmunoassay kits of iodine [^125^I] angiotensin I (lot number: 20140320), iodine [^125^I] angiotensin II (lot number: 20140320), and iodine [^125^I] aldosterone (lot number: 20140320) were provided by Beijing North Biotechnology Institute (Beijing, China). Morris water maze system was purchased from Chengdu Techman Technology Co., Ltd. (Chengdu, Sichuan, China). Terminal dexynucleotidyl transferase (TdT)-mediated dUTP nick end labeling (TUNEL) apoptosis detection kit was purchased from BOSTER Biotech Limited Company (Wuhan, Hubei, China).

### Experimental animals

Ten to 12-week-old healthy specific pathogen free (SPF) grade male Sprague Dawley (SD) rats (body weight 200 ± 10 g; Certificate No. SCXK2013-24) were provided by SPF Experimental Animal Medical Center, Southwest Medical University (Luzhou, Sichuan, China). All animal experiments were performed strictly in accordance with institutional guidelines and were approved (Permit No. 250114) by the Committee on Use and Care of Animals of Southwest Medical University (Luzhou, Sichuan, China).

### Preparation of the rat model of CCI

The rat model of CCI was modified from the method of simultaneous ligation of bilateral common carotid arteries of rats as previously described (Fujishima et al., 1976). Briefly, the rats were fasted overnight, weighed, and anesthetized with 1% sodium pentobarbital (30 mg/kg, intraperitoneal injection). The skin in the middle of the neck was incised and surrounding tissues were bluntly dissected, then, the common carotid arteries were ligated about 3–4 cm with moderate force at different times (day 1, 3, 7, 14, 21, and 30), the right common carotid artery was ligated 3 days after the ligation of left common carotid artery. Double ligation was implemented to ensure that the ligation was stable to block blood flow. During the ligation, the nerves must be separated completely and not be ligated. Excessive force must be avoided to prevent the breakage of the common carotid arteries. Eight rats were used for each group.

### Drug administration

Rats were orally administered with aliskiren (30 mg/kg/day), enalapril (4 mg/kg/day), candesartan (2 mg/kg/day), or the same volume of distilled water (DW) as control by gavage once a day for 30 consecutive days after surgery, while the rats without ligation were given DW as normal control. The doses of aliskiren, enalapril and candesartan were selected according to the ratio of body surface area to body weight per kg divided 60. The appropriate concentrations of drugs were prepared with DW and administered by oral gavage according to 1 ml/100 g (volume/body weight). Eight rats were used for each group.

### Specimen collection and parameters detection

Rats (eight for each group) were fasted overnight after drug or DW administration, then weighed and anesthetized with 1% sodium pentobarbital (30 mg/kg, volume: 0.3 ml/100 g, volume/body weight). Blood was taken from the abdominal aorta and placed in prepared anticoagulant vacuum blood collection tubes, then centrifuged at 3,000 × g for 10 min at 4°C. The plasma was collected and stored at −20°C for further analyses. After taking the blood, the brains were taken by decollation, the left side of cerebral cortex and whole hippocampus were separated immediately, weighed, and prepared as 10% homogenate at a ratio of 1:10 as tissue weight (g) to saline (ml), centrifuged at 3,000 × g for 10 min at 4°C, the supernatants were collected and stored at −20°C for further analysis. The parameters of plasma and tissue homogenates were determined by radioimmunoassay kits according to the manufacturer's instruction. The renin activity was determined as the production rate of angiotensin I in the plasma and calculated as the formation of renin activity = the concentration of angiotensin I of the tested sample at 37°C—the concentration of angiotensin I of the control sample at 4°C)/incubation time.

### Morris water maze test

Morris water maze was used for the study of spatial learning and memory of rats on day 30 after common carotid arteries ligation and drug administration and the general testing procedure was previously described (Morris, 1984; Barnhart et al., 2015). Briefly, the test was conducted in a round white pool (94 cm in diameter and 31 cm deeps) filled with water (30 cm depth, ~25°C). The escape platform was a 25-cm^2^ Plexiglas square, placed in the center of one quadrant of the pool, 15 cm from the pool's edge and submerged 1 cm beneath the water surface.

Navigation Test: The rats were gently placed into the water facing the pool wall with 4 trials for 120 s per trial with 30 min interval between the trials daily for 1 week, and the swimming track and escape latency period from the time entering the water to the time finding the platform were recorded by an online image video tracking system. The rats were dried with a dry towel to prevent the stress caused by low temperature after each experiment. The duration of escape latency was 120 s each time. The rat was discarded if it failed to find the platform within 120 s.

Space Exploration test: After the navigation test, the platform was removed; then rats were put into the pool from the point opposite to the quadrant of the platform, and the swimming track and number of times passing through the original platform within 120 s were recorded by the online image video tracking system.

### Apoptosis analysis of the hippocampus in rats by TUNEL assay

The apoptotic study was carried out on the paraffin sections of hippocampus of rats with TUNEL staining as previously described (Fang et al., 2005) after rats were orally treated with aliskiren (30 mg/kg/day), enalapril (4 mg/kg/day), candesartan (2 mg/kg/day), or DW (control) daily for 30 days. Briefly, the rats were anesthetized with 1.0% pentobarbital 12 h after the last dose of DW or drug treatment. Then, the tissues of hippocampus were taken and immediately fixed in 10% neutral buffered formalin, dehydrated, and embedded in paraffin sections for apoptotic analysis used TUNEL apoptosis detection kit following manufacturer's instruction. The apoptotic cells showed brown color in the nuclei of cells (normal cells as blue color). The integral optical density (IOD) was analyzed with Image-Pro® Plus Version 6.0 system (Media Cybemetics Inc., Silver Spring, MD, USA) as a parameter for quantification of apoptotic cells.

### Statistical analysis

All data were analyzed by IBM SPSS17.0 statistics software (SPSS Inc., Chicago, IL, USA) and results were expressed as mean ± standard deviation (*SD*). One-way univariate analysis of variance (ANOVA) was performed for the levels of renin, Ang II and ALD in the plasma, cerebral cortex and hippocampus of rats and repeated ANOVA was performed for the results of Morris water maze test and apoptosis analysis. A difference at *p* < 0.05 was considered to be statistically significant (marked as ^*^).

## Results

### Dynamic changes in the RAAS in CCI rats

**Figure 1:**
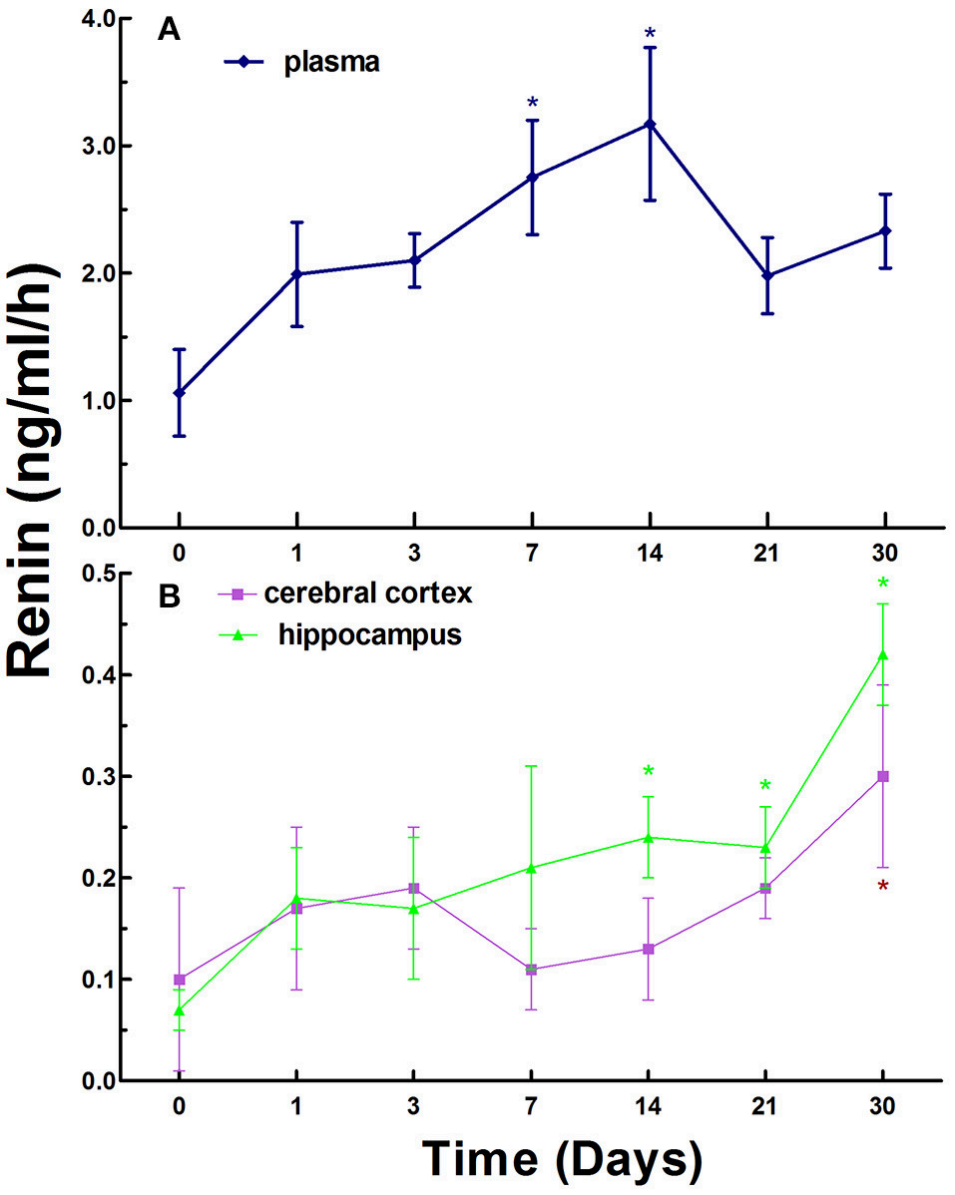
Renin activity in the plasma **(A)**, cerebral cortex and hippocampus **(B)** in normal and chronic cerebral ischemia (CCI) rats. There were eight rats used for each experimental group and expressed as mean ± SD. ^*^p < 0.05 vs. CCI rats treated with distill water (DW) by One-way univariate analysis of variance (ANOVA).

To investigate the dynamic changes in the RAAS in CCI rats, we first measured the levels of renin, Ang II, and ALD in the plasma and tissue homogenates of left side of cerebral cortex and whole hippocampus of normal rats (as control) and CCI rats established by surgical ligation of bilateral common carotid arteries at various times. The data in Figure 1A show that the renin levels in the plasma in normal rats (control) were 1.06 ± 1.11 ng/ml/h, the levels in plasma in CCI rats were generally increased compared to that of the control on day 1, 3, 7, 14, 21, and 30. There were statistically significant differences compared to the control (*p* < 0.05) on day 7 and 14.

The renin levels were 0.10 ± 0.11 and 0.07 ± 0.02 ng/ml/h in the cerebral cortex and hippocampus in normal rats, respectively. However, the levels in the cerebral cortex and hippocampus in CCI rats were notably increased compared to normal rats on day 1, 3, 7, 14, 21, and 30. There was a statistically significant difference (*p* < 0.05) in the cerebral cortex on day 30 only and in the hippocampus on day 14, 21, and 30 (highest) between normal rats and CCI rats (Figure 1B).

Interestingly, the overall levels of renin exhibited a trend with gradual increase over time in the plasma, cerebral cortex and hippocampus in CCI rats.

**Figure 2:**
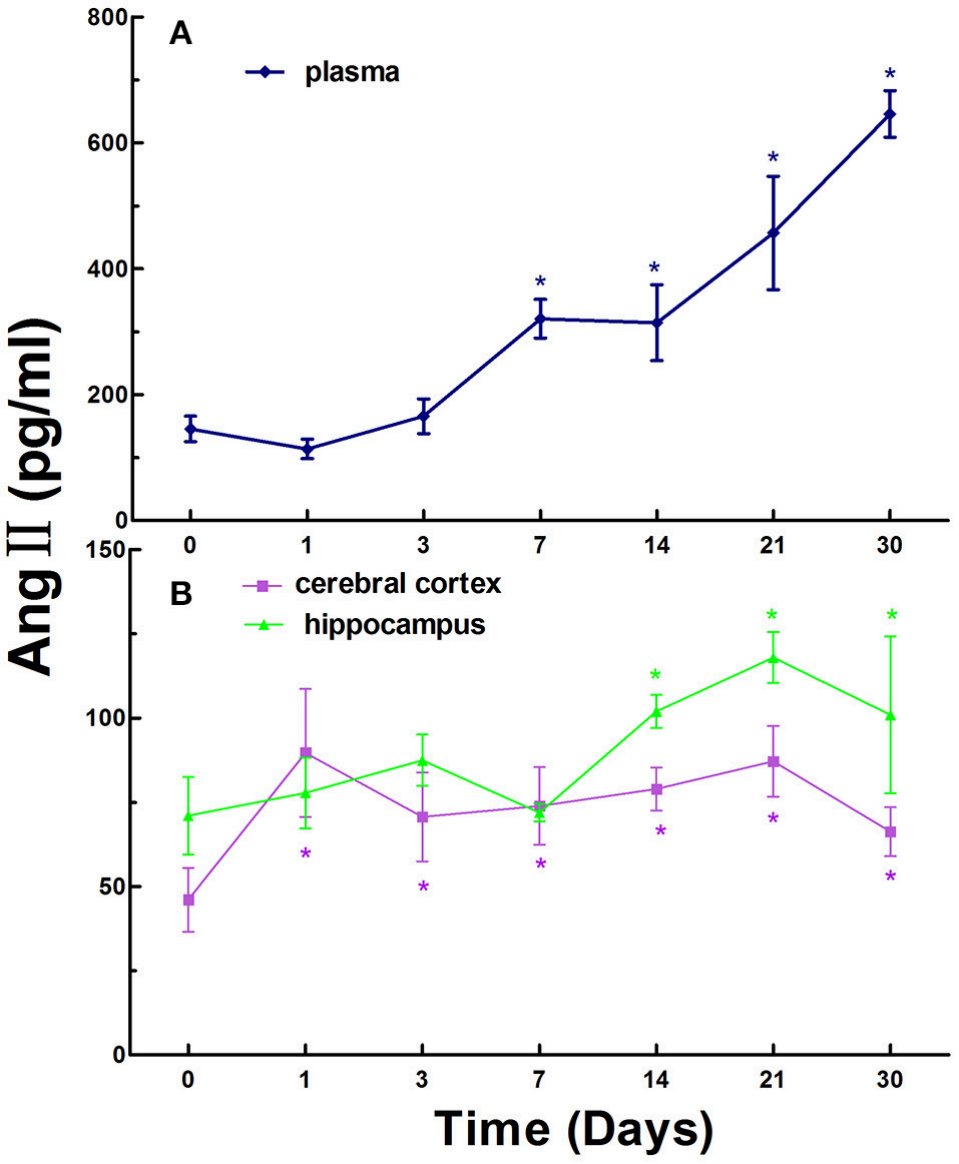
The levels of Ang II in the plasma **(A)**, cerebral cortex and hippocampus **(B)** in normal and chronic cerebral ischemia (CCI) rats. There were eight rats used for each experimental group and expressed as mean ± SD. ^*^p < 0.05 vs. CCI rats treated with distill water (DW) by One-way univariate analysis of variance (ANOVA).

Next, we investigated the levels of Ang II in the blood, cerebral cortex, and hippocampus in normal rats and CCI rats at different times. Ang II levels were 145.47 ± 66.05 pg/ml in the plasma in normal rats, while the levels of Ang II in the plasma in CCI rats were significantly increased compared to that of the control with statistically significant differences (*p* < 0.05) on day 7, 14, 21, and 30 (Figure 2A).

The Ang II levels were 46.03 ± 9.48 and 70.99 ± 11.46 pg/ml in the cerebral cortex and hippocampus in normal rats, respectively. The levels of Ang II in the cerebral cortex and hippocampus in CCI rats were markedly increased compared to that of the normal rats on day 1, 3, 7, 14, 21, and 30. There were statistically significant differences (*p* < 0.05) at every time point in the cerebral cortex and on day 14, 21, and 30 in the hippocampus in CCI rats compared to that of the control (Figure 2B).

Similarly, like the trend of renin, the overall levels of Ang II exhibited an increasing trend over time in the plasma, cerebral cortex and hippocampus in CCI rats (Figure 2).

**Figure 3:**
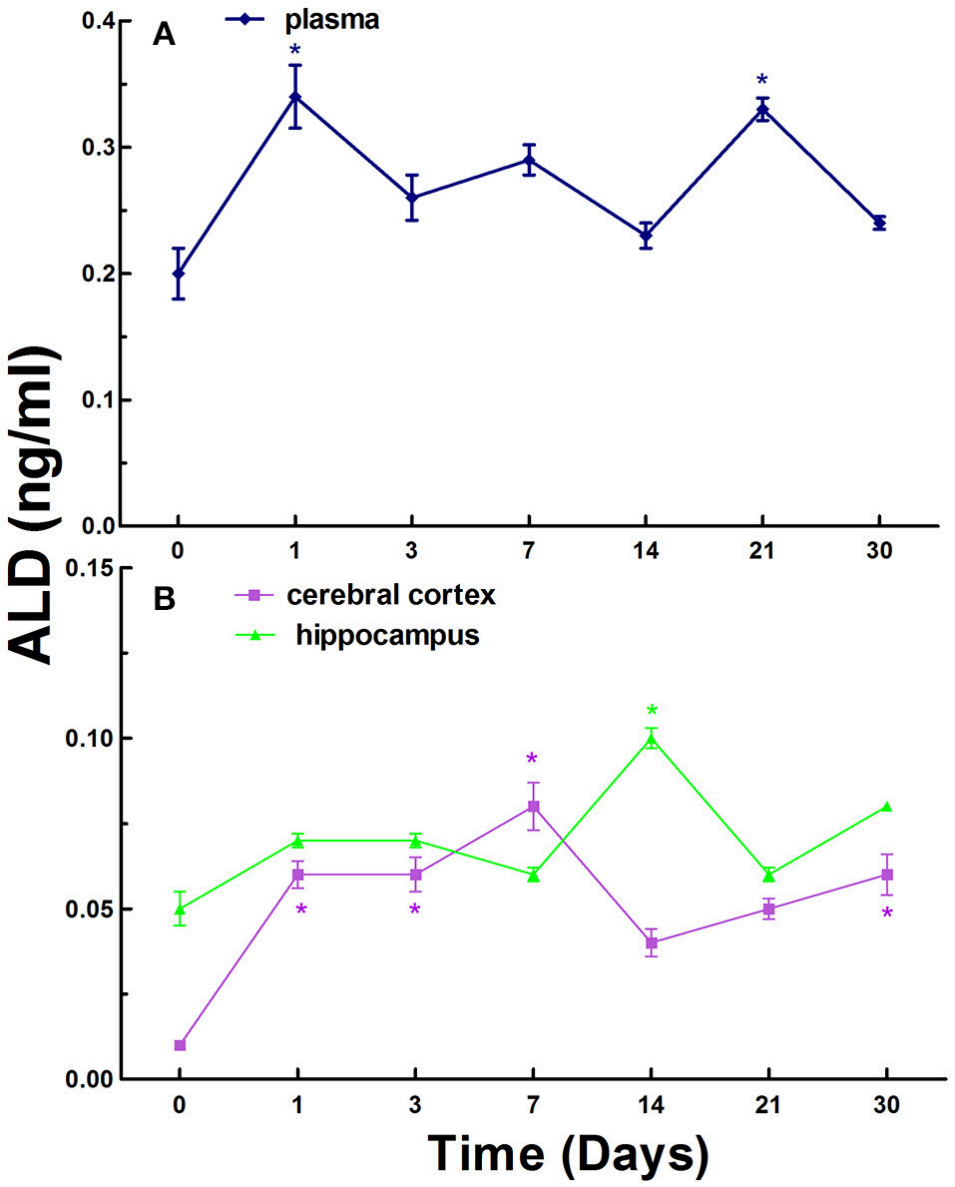
The levels of ALD in the plasma **(A)**, cerebral cortex and hippocampus **(B)** in normal and chronic cerebral ischemia (CCI) rats. There were eight rats used for each experimental group and expressed as mean ± SD. ^*^p < 0.05 vs. CCI rats treated with distill water (DW) by One-way univariate analysis of variance (ANOVA).

Finally, we investigated the levels of ALD in the blood, cerebral cortex, and hippocampus in normal rats and CCI rats at different times. The ALD levels were 0.20 ± 0.08 ng/ml in the plasma in normal rats, while the levels in the plasma in CCI rats were generally increased compared to that of the control on day 1, 3, 7, 14, 21, and 30. There were statistically significant increase (*p* < 0.05) compared to that of the control on day 1 and 21 (Figure 3A).

The ALD levels were 0.01 ± 0.02 and 0.05 ± 0.05 ng/ml in the cerebral cortex and hippocampus in normal rats, respectively. The levels of ALD in the cerebral cortex and hippocampus in CCI rats were also generally increased compared to control on day 1, 3, 7, 14, 21, and 30 (Figure 3B). There were significant increases (*p* < 0.05) on day 1, 3, 7, and 30 in the cerebral cortex in CCI rats compared to control. However, there was significant increase (*p* < 0.05) on day 14 only in the hippocampus in CCI rats compared to normal rats (Figure 3B).

In contrast to the levels of renin and Ang II, the overall levels of ALD exhibited relative fluctuations over time in the blood, cerebral cortex, and hippocampus in CCI rats (Figure 3).

### Effects of RAAS inhibitors on renin, Ang II, and ALD in the blood, cerebral cortex, and hippocampus in CCI rats

**Figure 4:**
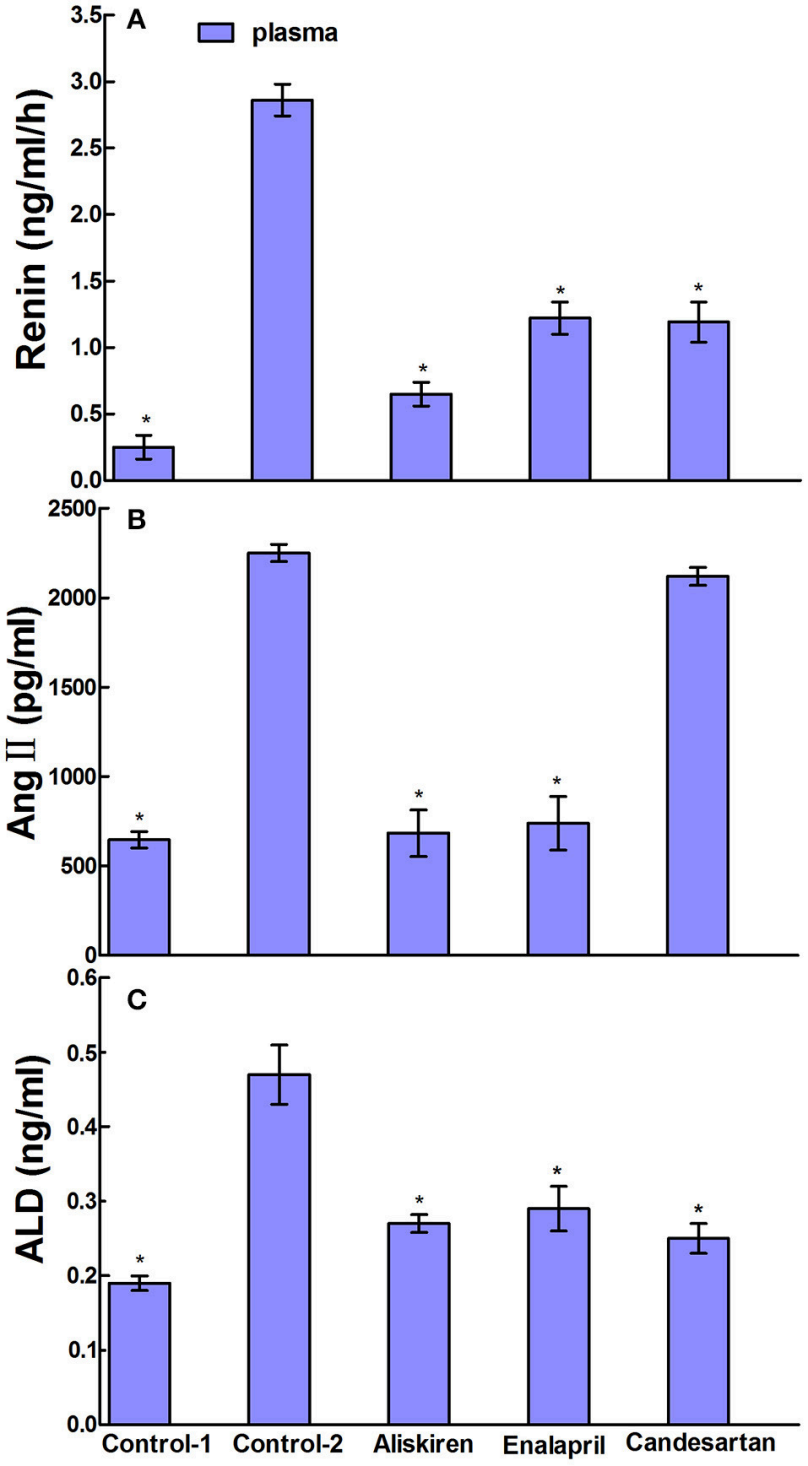
Effects of Aliskiren, Enalapril, and candesartan on the levels of renin activities **(A)**, Ang II **(B)**, and ALD **(C)** in the plasma in rats with chronic cerebral ischemia (CCI). CCI rats were orally administered with aliskiren (30 mg/kg/day), enalapril (4 mg/kg/day), candesartan (2 mg/kg/day), or same volume of distilled water (DW) as control-2 by gavage once a day for 30 consecutive days, while normal rats were given DW as control-1. There were eight rats used for each experimental group and expressed as mean ± SD. ^*^p < 0.05 vs. CCI rats treated with DW by One-way univariate analysis of variance (ANOVA).

**Figure 5:**
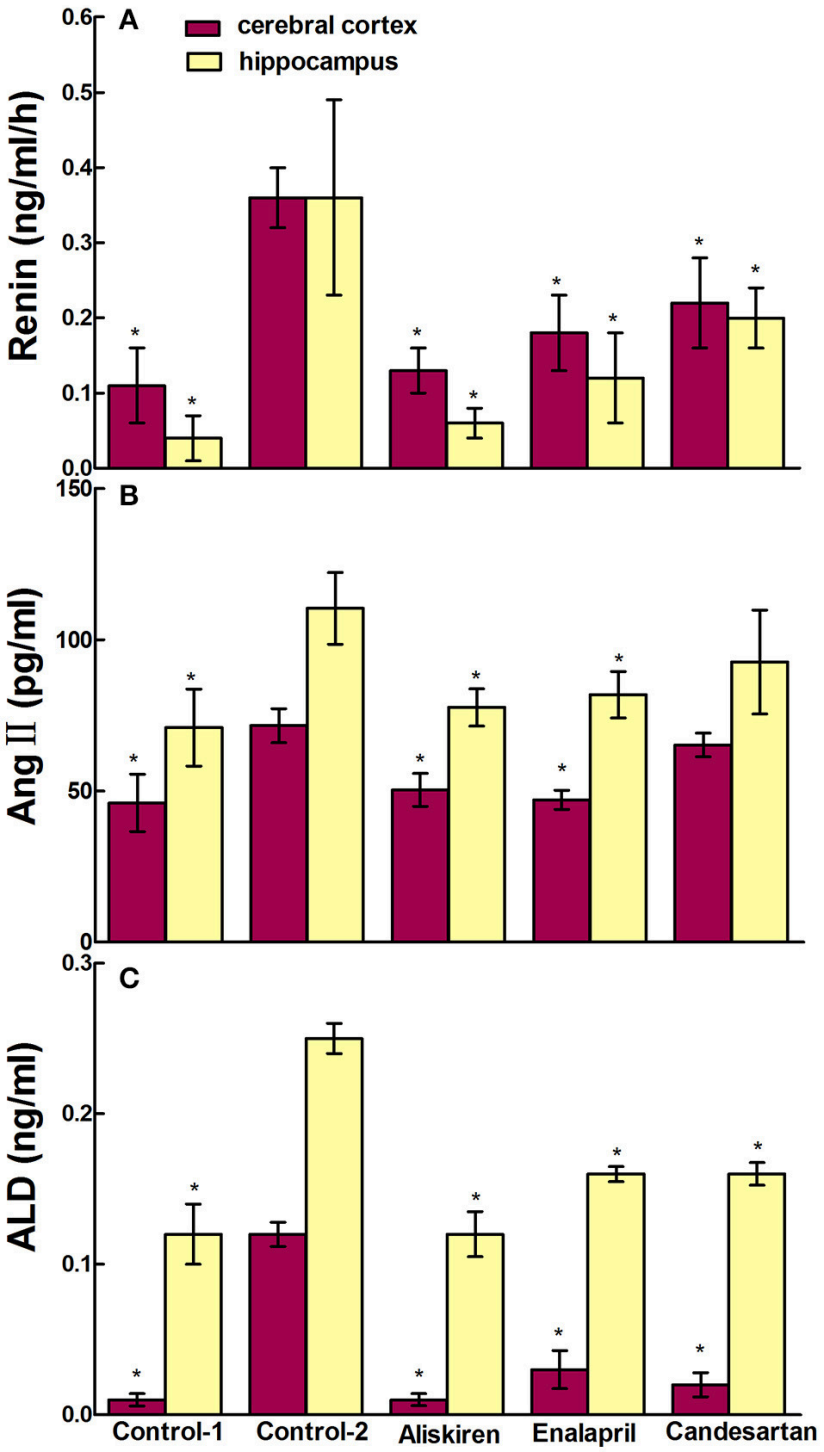
Effects of Aliskiren, Enalapril, and candesartan on the levels of renin activities **(A)**, Ang II **(B)**, and ALD **(C)** in the cerebral cortex and hippocampus in rats with chronic cerebral ischemia (CCI). CCI rats were orally administered with aliskiren (30 mg/kg/day), enalapril (4 mg/kg/day), candesartan (2 mg/kg/day), or same volume of distilled water (DW) as control-2 by gavage once a day for 30 consecutive days, while normal rats were given DW as control-1. There were eight rats used for each experimental group and expressed as mean ± SD. ^*^p < 0.05 vs. CCI rats treated with DW by One-way univariate analysis of variance (ANOVA).

RAAS regulates the physiological and pathological activities in the brain and plays a crucial role in the state of CCI in brain. Therefore, we studied the effects of RAAS inhibitors on renin, Ang II, and ALD in the blood, cerebral cortex, and hippocampus in CCI rats. First, we evaluated the effects of aliskiren, enalapril, and candesartan on renin activity in the CCI rats on day 30 after common carotid arteries ligation and compared to that of DW treatment and normal rats treated with DW. The data in Figure 4A show that the renin activity in the plasma was 0.25 ± 0.31 ng/ml/h in normal rats treated with DW and 2.86 ± 0.61 ng/ml/h in CCI rats treated with DW, which was significantly higher (*p* < 0.05) than that of normal rats. Interestingly, the renin activity was significantly decreased (*p* < 0.05) to 0.65 ± 0.37, 1.22 ± 0.40, and 1.19 ± 0.45 ng/ml/h, respectively, in the plasma in CCI rats treated with aliskiren, enalapril, or candesartan compared to that of DW treatment. The renin activity was 0.11 ± 0.06 and 0.36 ± 0.08 ng/ml/h, respectively, in the cerebral cortex in normal rats and CCI rats treated with DW, which was significantly different (*p* < 0.05) between the two groups. However, the renin activity was 0.13 ± 0.06, 0.18 ± 0.10, and 0.22 ± 0.11 ng/ml/h, respectively in the cerebral cortex in CCI rats treated with aliskiren, enalapril, or candesartan, which was significantly decreased (*p* < 0.05) compared to that of DW treatment (Figure 5A). Similarly, the renin activity in the hippocampus in CCI rats was also significantly higher (*p* < 0.05) compared to that of the normal rats as 0.36 ± 0.26 vs. 0.04 ± 0.03 ng/ml/h. However, the renin activity was 0.06 ± 0.02, 0.12 ± 0.06, and 0.20 ± 0.12 ng/ml/h, respectively, in the hippocampus in CCI rats treated with aliskiren, enalapril, or candesartan, which was significantly decreased (*p* < 0.05) compared to DW treatment (Figure 5A). The data indicate that aliskiren, enalapril, and candesartan can effectively decrease the elevated renin activity in the blood, cerebral cortex, and hippocampus in CCI rats, while aliskiren is the most effective among the three RAAS inhibitors.

Next, we evaluated the effects of aliskiren, enalapril, and candesartan on the levels of Ang II in CCI rats on day 30 after common carotid arteries ligation and compared to that of CCI rats and normal rats treated with DW. The Ang II levels were 2250.66 ± 103.97 pg/ml in the plasma in CCI rats treated with DW and was significantly higher (*p* < 0.05) than that of normal rats treated with DW (646.00 ± 97.50 pg/ml). Interestingly, the levels of Ang II were also significantly decreased (*p* < 0.05) to 683.40 ± 474.68 and 738.58 ± 577.99 pg/ml in the plasma in CCI rats treated with aliskiren or enalapril. However, the level of Ang II was 2120.49 ± 131.59 pg/ml in the plasma after candesartan treatment that was significantly higher (*p* < 0.05) than that of aliskiren or enalapril treatment but no significant different (*p* > 0.05) compared to DW treatment in CCI rats (Figure 4B). The levels of Ang II in the cerebral cortex and hippocampus in CCI rats treated with DW were also significantly higher (*p* < 0.05) than that of normal rats treated with DW (71.59 ± 5.61 vs. 46.03 ± 9.48 pg/ml and 110.36 ± 11.84 vs. 70.95 ± 12.76 pg/ml, respectively). Similarly, the Ang II levels were significantly decreased (*p* < 0.05) to 50.27 ± 5.44 and 77.60 ± 6.18 pg/ml, 47.06 ± 3.12 and 81.81 ± 7.71 pg/ml, in the cerebral cortex and hippocampus in CCI rats treated with aliskiren or enalapril compared to DW treatment. Once again, the Ang II levels was 65.21 ± 3.89 pg/ml in the cerebral cortex and 102.28 ± 8.65 pg/ml in the hippocampus in CCI rats treated with candesartan, that was significantly higher (*p* < 0.05) than that of aliskiren or enalapril treatment but no significant different (*p* > 0.05) compared to DW treatment (Figure 5B).

Finally, we evaluated the effects of aliskiren, enalapril, and candesartan on the levels of ALD in CCI rats on day 30 after common carotid arteries ligation and compared to that of CCI rats and normal rats treated with DW. The levels of ALD were significantly higher (*p* < 0.05) in the plasma in CCI rats treated with DW than that of the normal rats treated with DW (0.47 ± 0.21 vs. 0.19 ± 0.04 ng/ml). However, the ALD levels were significantly decreased (*p* < 0.05 vs. DW treatment) to 0.27 ± 0.03, 0.29 ± 0.05, and 0.25 ± 0.08 ng/ml, respectively in the plasma after the treatment of aliskiren, enalapril, or candesartan in CCI rats (Figure 4C). The ALD levels were 0.12 ± 0.02 and 0.25 ± 0.04 ng/ml in the cerebral cortex and the hippocampus in CCI rats, respectively, that was significantly higher (*p* < 0.05) than that of the normal rats treated with DW (0.01 ± 0.02 and 0.12 ± 0.08 ng/ml, respectively). However, the ALD levels were significantly decreased (*p* < 0.05 vs. DW treatment) to 0.01 ± 0.01 and 0.12 ± 0.06 ng/ml, 0.03 ± 0.03 and 0.16 ± 0.02 ng/ml, and 0.02 ± 0.02 and 0.16 ± 0.03 ng/ml in the cerebral cortex and the hippocampus, respectively, in CCI rats treated with aliskiren, enalapril, or candesartan (Figure 5C).

### Effect of RAAS inhibitors on spatial learning and memory in CCI rats by morris water maze test

**Figure 6:**
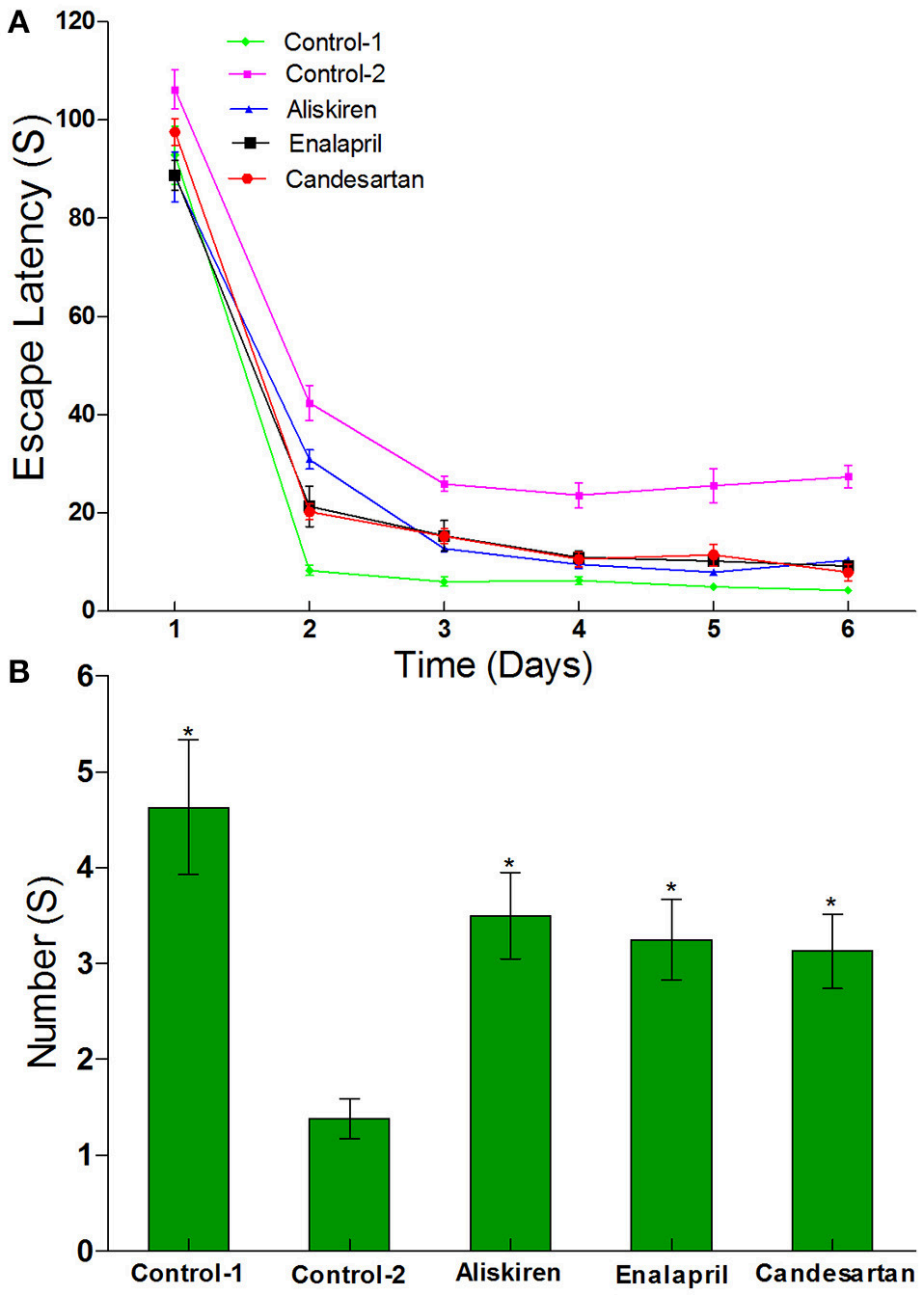
Effects of aliskiren, enalapril, and candesartan on the escape latency **(A)** and the number across the platform **(B)** in rats with chronic cerebral ischemia (CCI) by navigation and space exploration tests. CCI rats were orally administered with aliskiren (30 mg/kg/day), enalapril (4 mg/kg/day), candesartan (2 mg/kg/day), or same volume of distilled water (DW) as control-2 by gavage once a day for 30 consecutive days, while normal rats were given DW as control-1. There were eight rats used for each experimental group and expressed as mean ± SD. ^*^p < 0.05 vs. CCI rats treated with DW by repeated univariate analysis of variance (ANOVA).

Morris water maze test is a widely used method for assessing spatial learning and memory (Morris, 1984; Barnhart et al., 2015). First, the Navigation test was performed in normal rats (control) and CCI rats to investigate escape latency in the rats and the results are illustrated in Figure 6A. Results show that the escape latency periods were 27.40 ± 19.25 s in the CCI rats treated with DW on day 30 after common carotid arteries ligation and 4.28 ± 0.57 s in the normal rats treated with DW. There was significantly longer (*p* < 0.05) in the escape latency periods in CCI rats compared to that of normal rats, suggested that the cognitive function of CCI rats was obviously impaired. However, treatment of RAAS inhibitors has significantly shortened (*p* < 0.05) the escape latency periods compared to that of DW treatment in CCI rats. The escape latency periods were 10.39 ± 5.51, 9.25 ± 3.01, and 7.93 ± 2.70 s in the CCI rats treated with aliskiren, enalapril, or candesartan, respectively.

Next, the space exploration test was also performed to further study the cognitive function in CCI rats compared to that of normal rats and the results are presented in Figure 6B. The data show that the numbers of time passing through the platform were 1.38 ± 0.92 times in CCI rats treated with DW and 4.63 ± 2.45 times in normal rats treated with DW on day 30 after common carotid arteries ligation, which was significantly different (*p* < 0.05) between the two groups. However, the numbers of time passing through the platform were significantly increased (*p* < 0.05) to 3.50 ± 1.60, 3.25 ± 1.39, and 3.13 ± 1.13 times, respectively, in CCI rats treated with aliskiren, enalapril, or candesartan. The results suggest that RAAS inhibitors (aliskiren, enalapril, and candesartan) can effectively improve spatial learning and memory in CCI rats.

### Effect of RAAS inhibitors on apoptosis in the hippocampus in CCI rats

**Figure 7:**
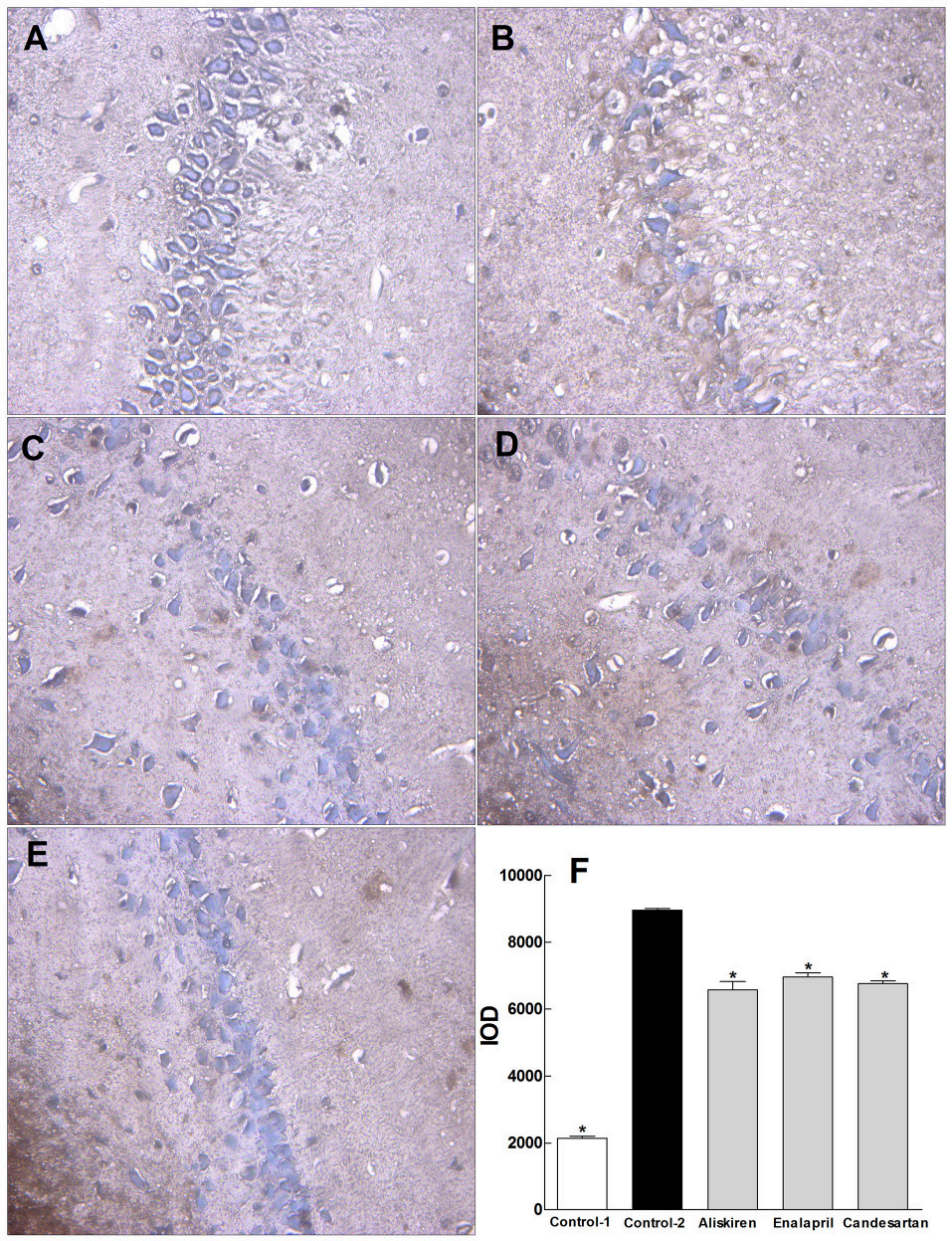
Effects of aliskiren, enalapril, and candesartan on apoptosis in the hippocampus in rats with chronic cerebral ischemia (CCI). **(A)** Hippocampal cells in a representative normal rat treated with distilled water (DW). **(B)** Hippocampal cells in a representative CCI rat treated with DW. **(C)** Hippocampal cells in a representative CCI rat treated with aliskiren 30 mg/kg/day. **(D)** Hippocampal cells in a representative CCI rat treated with enalapril 4 mg/kg/day. **(E)** Hippocampal cells in a representative CCI rat treated with candesartan 2 mg/kg/day. **(F)** The values of integral optical density (IOD) in normal rats treated with DW, CCI rats treated with DW, aliskiren (30 mg/kg/day), enalapril (4 mg/kg/day), or candesartan (2 mg/kg/day). Rats were orally administered with aliskiren, enalapril, candesartan or same volume of DW by gavage once a day for 30 consecutive days, then, anesthetized with 1.0% pentobarbital 12 h after the last treatment. The hippocampus was taken and immediately fixed in 10% neutral buffered formalin, dehydrated, and embedded in paraffin for apoptotic analysis by TUNEL apoptosis detection kit. The nuclei were colored blue in normal cells and brown in apoptotic cells. IOD was analyzed with Image-Pro® Plus Version 6.0 system as a parameter for quantification of apoptotic cells. There were eight rats used for each experimental group and expressed as mean ± SD. ^*^p < 0.05 vs. CCI rats treated with DW by repeated univariate analysis of variance (ANOVA).

To investigate the possible mechanism of RAAS inhibitors on improvement of spatial learning and memory and brain injury, we further studied the effect of aliskiren, enalapril, and candesartan on apoptosis in the sections of hippocampus in CCI rats by TUNEL immunohistochemical assays. The representative photomicrographs are shown in Figures 7A–E. The hippocampal cells show characteristic preserved normal histological features, which the cells arranged closely and orderly with blue-stained large and round nuclei, a few spontaneous apoptotic cells are clearly observed as brown and sparsely scattered in the viable portions of the hippocampal section in control rat treated with DW (Figure 7A). The hippocampal cells display loose arrangement with reduced numbers and irregular morphology, higher levels of apoptosis were detected in the hippocampal section in CCI rat treated with DW (Figure 7B). However, the numbers of apoptotic cells are partially but significantly decreased in the hippocampal sections in CCI rats treated with aliskiren, enalapril, or candesartan, although the cell numbers were still decreased compared to normal rat (Figures 7C–E). IOD was used as a parameter for quantification of apoptotic cells. The values of IOD in control rats treated with DW and CCI rats treated with DW, aliskiren, enalapril, or candesartan are summarized in Figure 7F. The value of IOD was 2140.77 ± 61.39 in control rats, but the value was much higher (*p* < 0.05) in CCI rats treated with DW than that of the control and reaching to 8958.97 ± 55.92. However, the values of IOD were dropped (*p* < 0.05 vs. DW treatment) to 6576.26 ± 241.48, 6958.50% ± 123.76, and 6757.87 ± 87.78, respectively, in CCI rats treated with aliskiren, enalapril, or candesartan. The data indicate that apoptosis plays important role in CCI and RAAS inhibitors can effectively inhibit apoptosis in the hippocampus in CCI rats.

## Discussion

RAAS plays an important role in the regulation of blood pressure and physiopathological activities in the brain in animals and humans (Atlas, 2007; Hajjar et al., 2009). In the present study, we established a rat model of CCI and evaluated the time course of renin, Ang II, and ALD in the blood, cerebral cortex, and hippocampus. The model was modified from the method of simultaneous ligation of bilateral common carotid arteries of rats (Fujishima et al., 1976) and it is easy to be produced with simple surgery, reproducible, reliable, low mortality, and convenience for long-term observation of time course for the studies of the dynamic changes of RAAS and cardiovascular diseases. Obvious changes of renin, Ang II, and ALD were observed in the blood compared to that of the cerebral cortex and hippocampus in CCI rats and the peak concentrations of renin, Ang II, and ALD in the blood appeared at different times, renin on day 14, Ang II on day 30, and ALD on day 21 (Figures 1A–3A). We also found that the main active substances (renin, Ang II, and ALD) of RAAS in CCI rats were significantly increased (*p* < 0.05) in the cerebral cortex and hippocampus compared to normal rats (Figures 1B–3B). The data indicate that the changes of main active substances of RAAS in the cerebral cortex and hippocampus in CCI rats may be the active risk factors for cerebral ischemia injury. In addition, the changes of main active substances of RAAS in the plasma were inconsistent with that of cerebral cortex and hippocampus, which may be an independent factor (Figures 1–3).

Aliskiren is a direct rennin inhibitor which blocks the RAAS at the first stage, decreases the activity of rennin, prevents the conversion of angiotensinogen to Ang and reduces the generation of Ang II and ALD to execute its role for the treatment of hypertension (Anderson, 2007; Politi et al., 2011). Studies have shown that aliskiren could enhance the fibrinolysis and anti-platelet aggregation, thereby blocking the formation of venous thrombosis and relieving the cerebral ischemia injury (Hermanowicz et al., 2013). It has also been reported that aliskiren decreased brain damage and improved memory deficits in CCI mice via the inhibition of oxidative stress (Dong et al., 2011). Enalapril is an ACE inhibitor which inhibits Ang I converted to Ang II to decrease Ang II level and leading to less vasoconstriction and lower blood pressure (Ferguson et al., 1982; Sweet et al., 1983). Studies have shown that enalapril could reduce oxidative stress and central energy metabolism, improve cerebral circulation, and prevent the decline of the ability in learning and memory in rats with brain injury (Nautiyal et al., 2013). Another study also showed that enalapril significantly augmented anti-oxidant activity in ischemic brain tissues by increasing glutathione (GSH) concentrations and attenuating the elevated level of malondialdehyde (MDA), and decreased brain oedema formation in response to ischemia, therefore, protecting blood-brain barrier (BBB) function and reducing neurological deficit score (Panahpour et al., 2014). Candesartan is a selective Ang II AT1 receptor antagonist and it binds to vascular smooth muscle Ang II AT1 receptors to antagonize vasoconstrictive effect of Ang II and reduce vascular resistance (McClellan and Goa, 1998). Krikov and colleagues reported that candesartan displayed neuroprotective effect after focal ischemia by significantly reducing brain injury and improving neurological outcome, the effect was associated with increased activity of the neurotrophin BDNF/TrkB system (Krikov et al., 2008). It has been reported that candesartan has a significant effect on neuroprotection (Saavedra, 2012). Tota and colleagues showed that candesartan significantly improved spatial learning and memory in the mice with memory impairment induced by intracerebral streptozotocin (Tota et al., 2008). Thus, RAAS inhibitors not only could regulate blood pressure and water-electrolyte metabolism but also attenuate nerve cell damage after cerebral ischemia.

In the present study, we studied the effects of RAAS inhibitors enalapril, aliskiren, and candesartan on renin, Ang II, and ALD in the blood, cerebral cortex, and hippocampus in CCI rats on day 30 after common carotid arteries ligation and compared to that of DW treatment. The data showed that aliskiren, enalapril, and candesartan effectively decreased (*p* < 0.05) the significantly elevated levels of renin and ALD in the blood, cerebral cortex, and hippocampus in CCI rats. However, aliskiren and enalapril but not candesartan significantly decreased (*p* < 0.05) the elevated levels of Ang II in the blood, cerebral cortex, and hippocampus in CCI rats (Figures 4, 5). The data are consistent with the pharmacologic effect of candesartan as an Ang II AT1 receptor blocker. Interestingly, the results also showed that there were different levels of renin activity, Ang II, and ALD with the treatments of enalapril, aliskiren, or candesartan in CCI rats. The renin activity was higher (*p* < 0.05) in the rats treated with enalapril or candesartan than that of the rats treated with aliskiren; the Ang II level was higher in the rats treated with candesartan than that of the rats treated with aliskiren or enalapril; while the ALD level was similar and lower after the treatments of all three drugs in CCI rats (Figures 4, 5). The results indicate that the drug actions were associated with the regulation of RAAS feedback so the ALD levels were low after the treatment of enalapril, aliskiren, or candesartan because it is a downstream substance of the common reaction chain of RAAS.

RAAS regulates brain function and plays a crucial role in CCI (Saavedra, 2012; Hermanowicz et al., 2013; Nautiyal et al., 2013; Panahpour et al., 2014). Therefore, we also studied the protective effects of aliskiren, enalapril, and candesartan on cognitive function in CCI rats. Morris water maze test is a widely used method for assessment of spatial learning and memory (Morris, 1984; Barnhart et al., 2015). The tested results showed that spatial learning and memory of CCI rats on day 30 after common carotid arteries ligation were significantly decreased compared to that of normal rats, suggesting that cognitive functions were significantly impaired in CCI rats (Figure 6). The studies demonstrated that chronic ischemic induced brain injury and dementia have developed after 30 days CCI with related pathological changes in CCI rats (Huang et al., 2010, unpublished data). The results also showed that the intervention by different RAAS inhibitors could significantly relieve learning and memory impairment induced by cerebral ischemia in CCI rats (Figure 6). Although, there were different levels of renin, ALD, and particularly Ang II in the blood, cerebral cortex and hippocampus in CCI rats treated with enalapril, aliskiren, or candesartan, no significant difference was observed in the improvement of learning and memory among the CCI rats with different treatment.

Hippocampus plays an important role in spatial learning and memory while apoptosis is a key factor to induce brain injury in cerebral ischemia (Shrager et al., 2007; Broughton et al., 2009). Studies have shown that active ingredients such as flavonoids and saponins from natural products significantly improved spatial learning and memory and neuronal injury by inhibiting hippocampal apoptosis in CCI and AD rats (Cheng et al., 2012; Wang et al., 2017). Therefore, we investigated the effects of enalapril, aliskiren, and candesartan on hippocampal apoptosis in CCI rats for the possible mechanism in improvement of spatial learning and memory and brain injury. In the present study, we demonstrated that apoptotic cells were significantly increased (*p* < 0.05) in the hippocampus in CCI rats compared to that of normal rats and the three RAAS inhibitors can partially but significantly inhibit apoptosis induced by CCI in the hippocampus in CCI rats (Figure 7).

Taken together, our data have proved that the tested RAAS inhibitors not only significantly affect the dynamic changes of RAAS in the blood, cerebral cortex, and hippocampus but also markedly improve spatial learning and memory and inhibit hippocampal apoptosis in CCI rats (Figures 6, 7). Therefore, our studies are relevant to scientific research and clinical practice for RAAS inhibitors.

## Conclusion

In conclusion, we studied the dynamic changes in renin, Ang II, and ALD and the effects of RAAS inhibitors aliskiren, enalapril, and candesartan on the RAAS in the blood, cerebral cortex and hippocampus, spatial learning and memory and hippocampal apoptosis in a rat model of CCI. The levels of renin and Ang II were significantly higher in the plasma, cerebral cortex and hippocampus in CCI rats compared to normal rats. However, the levels of renin, Ang II and ALD were significantly decreased by aliskiren and enalapril, renin and ALD by candesartan in the blood, cerebral cortex and hippocampus in CCI rats. Spatial learning and memory were significantly decreased but hippocampal apoptosis was notably increased in CCI rats compared to that of normal rats and aliskiren, enalapril, and candesartan significantly improved spatial learning and memory and inhibited hippocampal apoptosis with equal effectiveness. The results demonstrate that the RAAS plays an important role in the occurrence and development of cerebral ischemia and RAAS inhibitors are not only effective in the treatment of hypertension but also significantly improve spatial learning and memory in CCI rats. The possible mechanism for the improvement of spatial learning and memory and brain injury may be associated with the inhibition of hippocampal apoptosis.

## Author contributions

XH and SC designed the experiments, analyzed the data, and wrote the manuscript; GzL, GcL, HL, BL, and JY performed the experiments. All authors discussed the results and contributed to the refinement of the manuscript.

### Conflict of interest statement

The authors declare that the research was conducted in the absence of any commercial or financial relationships that could be construed as a potential conflict of interest.
